# The Effect of Encapsulated Powder of Goji Berry (*Lycium barbarum*) on Growth and Survival of Probiotic Bacteria

**DOI:** 10.3390/microorganisms8010057

**Published:** 2019-12-28

**Authors:** Prodromos Skenderidis, Chrysanthi Mitsagga, Dimitrios Lampakis, Konstantinos Petrotos, Ioannis Giavasis

**Affiliations:** 1 Department of Biosystems Engineering/Agricultural Technology, University of Thessaly, 41110 Larissa, Greece; dlampakis@gmail.com (D.L.); petrotos@teilar.gr (K.P.); 2 Department of Food Technology, University of Thessaly, End of N. Temponera Street, 43100 Karditsa, Greece; mitsagga.chrisanthi@hotmail.com (C.M.); igiavasis@teilar.gr (I.G.)

**Keywords:** Goji berry extracts, probiotic, prebiotic, nutraceutical, polysaccharides, polyphenols, *Bifidobacterium*, *Lactobacillus*, encapsulation

## Abstract

The aim of the present work was to investigate the potential prebiotic action of Goji berry powder on selected probiotic bacteria grown in a nutritive synthetic substrate and in simulated gastric and intestinal juices. Different probiotic strains of *Bifidobacterium* and *Lactobacillus* were grown in these substrates with or without the addition of encapsulated goji berry extracts of different polysaccharide and polyphenol contents. The results proved that the addition of the extracts promoted the proliferation of probiotic strains and, in particular, increased the number of bacterial colonies of *Bifidobacterium animalis subsp. lactis* (Bb*12*), *Bifidobacterium longum* (Bb*46*), and *Lactobacillus casei* by 2, 0.26, and 1.34 (log cfu/mL), respectively. Furthermore, the prebiotic effect seems to be correlated to Goji berry polysaccharides and/or polyphenols, higher contents of which (under the tested concentrations) could increase the stress tolerance of *B. lactis* and *B. longum* in a simulated gastrointestinal environment. According to the findings of the present research, it can be suggested that the Goji berry encapsulated extracts could be used as prebiotic additives in food or nutraceuticals, in order to stimulate growth or protect the viability of probiotic strains of *Bifidobacterium* and *Lactobacillus*.

## 1. Introduction

The botanical kingdom offers endless opportunities to isolate new functional substances, such as those originating from various berries, like the Goji berry [1,2]. These substances may consist of polyunsaturated fatty acids, flavanols (anthocyanins, proanthocyanidins and quercetin) and ellagic acid, which exhibit antioxidant, antimicrobial, anti-inflammatory and anti-mutagenic properties [1,3,4,5,6].

*L. barbarum* (Goji berry) is of great importance as a functional food product and has been used for more than 2500 years for nutritional and medicinal purposes [7]. *L. barbarum,* is a solanaceous defoliated shrubbery native to Asian areas such as China and Tibet [8]. It is popular in traditional medicine in Asian countries due to its biofunctionality, which is related to various components that include polysaccharides, carotenoids, and phenolic compounds [9]. The ancient herbalist classics recorded that *L. barbarum* nourishes the liver and kidneys and brightens the eyes [9]. Recent studies have shown that *L. barbarum* exhibits neuroprotective and hepatoprotective properties, potential benefits against cardiovascular and inflammatory diseases, anti-diabetic action by improved control of glucose metabolism, anti-aging effects, cytoprotection and protection from memory deficiencies and lung disorders, and anticancer activity [10,11,12], thanks to which, *L. barbarum* fruit has recently gained increasing popularity in Europe and North America [12].

Recently Goji berries have been also positively evaluated for their prebiotic potential in foods like yogurt [13], since their polysaccharides may be selectively utilized by some probiotic bacteria [14], although a potential prebiotic effect may be also linked to other molecules like polyphenols, which may stimulate the growth of probiotic bacteria in the gut, or inhibit the growth of antagonistic bacteria in the complex intestinal microbiota [15].

Gut colonization by beneficial probiotic bacteria is recognized as an essential parameter for intestinal health, and human health in general. It occurs in early life, as *Bifidobacterium* and *Lactobacillus* species attach to the gastrointestinal tract, which is necessary for establishing the gut mucosal barrier, maturation and modulation of the immune system, preventing infections by enteric pathogens and improving gastrointestinal function, digestion, and metabolism [16,17,18,19,20]. Nowadays, the presence (or supplementation) of certain probiotic bacteria, prebiotics or symbiotics (mixed preparations of probiotics and prebiotics) in the gastrointestinal tract is linked to prevention or reduced risk of ulcer, gastroenteritis, inflammation, colon cancer and metabolic syndrome (the latter involving hypolipidemic, hypocholesterolemic and potential hypoglycemic activity) as well as preterm birth and neonatal gastrointestinal disorder [21,22,23,24,25,26,27,28,29]. However, gut microbiota are not stable throughout life, and significant changes can occur naturally throughout the life cycle, or be provoked by many factors, including a diet poor in plant fiber and prebiotics. Thus, foods that are rich in prebiotics (i.e., carbohydrates or other components that can be selectively metabolized by probiotic bacteria and alter the gastrointestinal microbiota in favor of the probiotic bacteria), are of great importance in the modern diet [18,30,31,32], although the effects of prebiotics, even in high doses, cannot be surpassed by a healthy, balanced diet [30]. Thus, novel probiotics, prebiotics, and synbiotics from different sources have been constantly sought after in recent decades, in order to form part of a fortified functional food for infants, children, or adults, or a food supplement for nutraceutical and therapeutic purposes [33,34,35,36]. Notably, the action of probiotics in the gut is generally shorter, in comparison to the action of prebiotics which can be traced for several weeks after their administration, and also, the administration of exogenous probiotics is usually a short-term treatment (e.g., after antibiotics administration) [32]. Therefore, the characterization and consumption of novel prebiotics as part of a healthy diet is of great importance, in order to induce the endogenous health-promoting gut microbiota in the long term.

In this respect, the aim of this research was to investigate the in vitro prebiotic potential activity of Goji berry fruit extracts with regard to potential stimulatory effect on the growth of probiotic bacteria (i.e., *Lactobacillus* and *Bifidobacterium* species) in nutritive synthetic substrate. Furthermore, the prophylactic effect towards probiotic bacteria in simulated gastric and intestinal juices was studied. 

## 2. Materials and Methods

### 2.1. Goji Berry Powder Preparation

Goji berries were extracted following optimal extraction conditions and the powder preparation was performed as described in our previous study [11]. It is well-known that freeze-dried carbohydrates and proteins may exist in an amorphous state with time-dependent physical properties that affect their storage stability [37]. This amorphous material state undergoes a change from a viscous rubbery state to a “glassy” state at the glass transition temperature (Tg). Thus, after the extraction, the liquid Goji berry extract was mixed with various quantities of maltodextrin DE18 (140, 70, and 20 mg/mL) before lyophilisation in order to avoid the negative glass transition effect on the obtained lyophilisation powders and to obtain a free-flowing powder. In the samples with the lower amount of added maltodextrin, solid silicon dioxide (SiO_2_) was also added in order to increase Tg and avoid the glass transition effect on the powder. The final dried and encapsulated extracts which were investigated for potential prebiotic activity are presented in Table 1.

### 2.2. Determination of Total Polyphenol Content (TPC) and Total Carbohydrate Content (TCC) of the Extracts

For the TPC determination, the method described by Skenderidis et al. [6] was used. Briefly, 20 µL of each extract was mixed with 1.58 mL water, and then with 100 µL of Folin–Ciocalteu reagent (0.2 N). Subsequently, 300 µL of Na_2_CO_3_ solution (200 g/L) was added and after two hours of incubation in the dark the absorbance was measured at 765 nm. TPC was calculated on the basis of the calibration curve of gallic acid and expressed as gallic acid equivalents (GAE) in mg mL^−1^ of extract.

TCC was determined using the phenol–sulphuric acid method [38]. Briefly, 0.2 mL of extracts solution was mixed with 0.2 mL of 5% phenol solution, followed by adding 1 mL of concentrated sulphuric acid and shaking the mixture for 30 min. The absorbance was measured at 490 nm and used to quantify polysaccharide, based on the standard curve of d-glucose, which was prepared by plotting six concentrations (0.01–1 mg/L) against their absorbance (*R^2^* = 0.999). The equation was obtained by linear regression: *y* = 6.0464*x* [6], where y is absorbance at 490 nm and *x* is the concentration of d-glucose (mg/L) in the standards. The extracts were further diluted to adjust concentration within the linear range of the standard curve, and the TCC was expressed in dry basis.

### 2.3. Sources and Cultivation of Probiotic Cultures

Different commercially available probiotic bacteria of the genus *Lactobacillus* and *Bifidobacterium* were used in this study. These included: *L. acidophilus* (Dupont™ Dαnisko®, Copenhagen, Denmark), *L. casei* (Christian Hansen Laboratory, Horsholm, Denmark), *L. rhamnosus* (Christian Hansen Laboratory), *B. Animalis* subsp. lactis (strain BB-12®, Christian Hansen Laboratory), and *B. longum* (strainBb-46®, Christian Hansen Laboratory), which were stored in frozen lyophilized form. Lactobacilli were cultured in MRS Broth (Acumedia, MI, USA) for 48 h at 37 °C, while Bifidobacteria were cultivated in TPY Broth (Conda, Madrit, Spain) anaerobically (by addition of 10 mL of sterile paraffin in the 100 mL bottles) for 48 h at 37 °C, before use. For all tested strains, the cells were centrifuged (4 °C, 4000× *g*, 30 min), washed twice with sterile saline solution, and resuspended in an equal volume of sterile saline before inoculation.

### 2.4. Growth on Nutritive Synthetic Substrate

Fresh cultures of each Lactobacillus strain were added at a ratio of 1% (volume of culture/final volume of growth medium) in sterile MRS Broth with 0.1% (*w/v*) addition of the examined Goji berry powder samples and incubated at 37 °C. The same procedure was followed for both Bifidobacteria species, except that they were grown anaerobically. Enumeration of cell population of each liquid growth medium was carried out on MRS agar (Oxoid, UK) for Lactobacilli and on TOS-Propionate Agar (base) with Bifidobacterium selective supplement (Conda S.A., Spain) for Bifidobacteria, which were growth anaerobically by the use of Anaerocult A in anaerobic jars (Merck, Germany), after a 72 h incubation at 37 °C for all probiotic strains.

The pH of the liquid growth medium was measured in triplicate (using a HANNA pH 210 pHmeter) and the viable total counts of Lactobacilli and Bifidobacteria (log cfu/mL) were determined at time intervals of 0, 8, 25 and 34 h of incubation in triplicate samples, from which the mean values and standard deviation were calculated. In every experiment a negative control without any added prebiotic (Goji berry) sample was included.

### 2.5. Study of Potential Prophylactic Effect of Goji Berry Powder Extracts towards Probiotic Species in a Simulated Gastrointestinal Environment

The method presented by Pak et al. [39] was followed, with some modifications described below, in order to study the protective effects of Goji berry powder extracts against a simulated gastrointestinal environment.

#### 2.5.1. Preparation of Simulated Gastric and Intestinal Juices

##### Gastric Juice

The simulated gastric juice was prepared by suspending of 0.22% (*w/v*, 1.2 U mg^−1^) pepsin (Sigma, St. Louis, MO, USA) in a 0.5% (*w/v*) NaCl solution (Penta, Prague, Czech) and adjusting the pH to 2 with 0.1 N HCl.

##### Intestinal Juice

The simulated intestinal juice contained 0.33% (*w/v*) porcine bile extract salts (Becton Dickinson, Franklin Lakes, NJ, USA) dissolved in distilled water. A total of 90 mL of the above juices were sterilized by filtration through 0.2 mm Whatman® membranes (Sigma, St. Louis, MO, USA), and mixed with 10 mL aqueous sterile Goji berry extract solution (1% *w/v*), in order to form a final concentration of 0.1% (*w/v*) of Goji berry powder in the final simulated juice. To this final broth, 1 mL (1% *v/v*) of each tested probiotic culture was aseptically added.

After inoculation, the simulated gastric and intestinal juices were incubated at 37 °C for 3 days and the population of viable bacteria was counted at 0, 1 and 3 h intervals (in the gastric juice) and 0-, 0.5-, and 1-h intervals (for the intestinal juice) during incubation. Here, 0 h corresponds to the sample taken as soon as the culture was inoculated into the gastric or intestinal juice. All measurements were made in triplicate and mean values and standard deviation for each measurement were calculated.

### 2.6. Statistical Analysis

A randomized complete block experiment design was selected for the study followed by a Fit General Model Analysis. Data are expressed as the means of 3 measurements. In order to measure the potential stimulation of bacterial growth in liquid growth media, the difference (increase or decrease) of the bacterial population (log cfu/mL) and culture pH (i.e., production of organic acids during growth) before and after the supplementation of Goji berry extract powder was estimated and expressed as mean delta value. Furthermore, the cell viability (log cfu/mL) was calculated in simulated gastrointestinal conditions using the same methodology. The mean delta log cfu/mL value resulted from the incubation of the probiotic bacteria with or without supplementation of Goji berry extracts powder. Standard deviation (SD) was calculated and the average values along with the SDs are documented in the respective tables. Statistical differences among the mean values were detected by multi-factorial ANOVA, focusing on the effect of sample type on microbial population. This was followed by the Tukey pairwise comparison test, while statistical significance was set at *p* ≤ 0.05 level. MiniTab®17.1.0 software (Minitab LLC., State College, PA, USA) was used as a tool to perform the statistical analyses.

## 3. Results and Discussion

The data of culture pH and bacterial growth (log cfu/mL) in the synthetic growth medium were collected at 8, 24 and 34 h after the initial inoculation (Table 2 and Table 3, respectively). The results shown in Table 2 suggest that *L. acidophilus* exhibits the highest lactate-producing activity, which was evident in the greatest pH reduction (from 6.5 to 3.63 after 34 h of incubation) among the five probiotic bacteria tested with or without encapsulated Goji berry extract addition. Also, lactate production and consequent pH reduction appears to be enhanced after the addition of sample 3 to *L. acidophilus* cultures, especially at 24 and 34 h (lower pH compared to the control by 0.52 and 0.20, respectively), and to lesser extent, after the addition of sample 1 (pH reduction by 0.5 compared to the control, at 8 h). *L. casei* and *L. rhamnosus* exhibited a similar trend of significant reduction of culture pH compared to the control, especially after the addition of samples 3 and 1, while the addition of sample 2 had a minor (not statistically significant) effect on stimulating lactate production in *L. casei* and *L. acidophilus*.

With regard to the two tested *Bifidobacterium* species, the addition of all Goji berry extract samples (1–3) led to a slight pH reduction in both species, but *B. longum* cultures seemed to produce significantly more organic acids at 24 and 34 h after the addition of Goji berry extracts, compared to the control, especially when sample No 3 was added to the culture, which resulted in a pH drop by 0.34 at 34 h compared to the control (Table 2). Organic acid production and pH reduction of the culture medium was only stimulated in *B. lactis* after the supplementation of sample 3. Overall, it appears that sample 3, which was the Goji berry extract with the highest amount of polysaccharides, as well as polyphenols, had the highest stimulatory effect upon organic acid production in all probiotic *Lactobacillus* and *Bifidobacterium* species tested (Table 2).

Apart from increased acid producing capacity, the (higher) reduction of pH of a probiotic culture may also indicate or correlate with an increased population. Indeed, by adding sample No 3 to *L. casei*, which had the highest population at 34 h among the 3 *Lactobacillus* species, a significant increase in the population was observed after 34 h of incubation, reaching a maximum difference of 1.34 log cfu/mL compared to the control at 34 h (Table 3). Samples 1 and 2 also enhanced the growth of this species at 34 h. A similar (but less pronounced) stimulation of growth at 34 h was evident in *L. acidophilus* cultures after the addition of samples 1, 2, and 3 (Table 3). In fact, it seems that the addition of the Goji berry extracts prolongs the growth or stationary phase of these two *Lactobacillus* species, compared to the control, which enters the death phase earlier (before 34 h), since the latter (control) has a significantly reduced population at 34 h compared to 24 h. On the other hand, the growth of *L. rhamnosus* was not significantly affected by the presence of Goji berry extracts, which only caused a minor (not significant) increase in the population at 24 h after the addition of samples 3, 1, and 2 (Table 2).

Between the two along *Bifidobacterium* species *B. lactis* showed the highest stimulation of growth after addition of the three Goji berry extracts, having 2.0, 1.8, and 1.75 log cfu/mL higher population compared to the control at 34 h, after the addition of samples 3, 2, and 1 respectively (Table 3). The growth of *B. longum* was less affected but was mainly favored by sample 3, at 34 h (increased by 0.26 log cfu/mL) and to a lesser extent by sample 1 (Table 3). These results indicate a potential stimulatory effect of the encapsulated Goji berry extracts, and especially sample 3, on the growth and viability of most *Lactobacillus* and *Bifidobacterium* tested here, which is crucial in exhibiting their probiotic properties.

According to Pavli et al. [40], in order for probiotic bacteria to provide their benefits to consumer’s health, they should remain viable during their transition to the gastrointestinal tract at population levels of 10^6^–10^7^ cfu/g. Some of the most important inhibitory factors against probiotic bacteria are the acidic environment and the effect of the proteolytic enzyme pepsin in the stomach. According to Ruiz et al. [41], the bacteriocidal activity of bile salts is another important factor that affects probiotic bacteria in the intestinal environment. In order to test further the viability and survival of probiotic strains in simulated gastric and intestinal conditions, and the potential protective effect of Goji berry extracts in those environments, the two *Bifidobacterium* species, along with *L. casei* (which had the highest stimulatory effect on growth after addition of Goji berry extract, among the three *Lactobacillus* species) were used.

In our study, *L. casei* survived well in a simulated gastric juice and was positively affected by the added Goji berry extracts, in a similar as *B. lactis*, while *B. longum* was less resistant to prolonged acidic conditions and action of pepsin in gastric juices, although it was favored greatly by the addition of Goji berry extracts (Table 4). More specifically, the control of *B. longum* experienced a sheer reduction from 8.84 to 4.7 log cfu/mL after 3 h in gastric juice, while addition of sample 3 preserved the viable population at 7.02 (2.32 log cfu/mL higher than the control) and samples 1 and 2 resulted in a lower, but still important improvement of survival after 3 h in gastric juice by 0.6 log cfu/mL (Table 4). *B. lactis* also benefited from the presence of samples 3, 1, and 2 in the gastric juice, since it had a higher survival compared to the control at 34 h, by 0.98, 0.67, and 0.63 log cfu/mL, respectively. *L. casei* population was only slightly increased by the addition of Goji berry extracts (by 0.18 and 0.16 log cfu/mL after the addition of samples 1 and 3, respectively), following 3 h of incubation (Table 4).

With regard to the survival in intestinal juice, *B. longum* exhibited the best survival under these conditions and the optimal protective effect of Goji berry extracts, especially sample 3, after 1 and 3 h of incubation, when it attained an increased population by 0.94 and 1.14 log cfu/mL compared to the control (Table 5). Samples 1 and 2 were also beneficial for its survival but to a lesser extent. Similarly, *B. lactis* retained a higher population by ~0.8 log cfu/mL at 1 and 3 h of incubation in intestinal juice supplemented with sample3, while sample 1 and 2 had a less important protective effect at 3 h (Table 5). *L. casei* was severely affected by the bile salts of the intestinal juices at 1h and especially at 3h of incubation. Even though the population fell below the detection limit at 3 h, there was a significant protective effect in cell viability at 1 h, after the supplementation of samples 3, 1, and 2 (with a decreasing order of significance), showing that Goji berry extracts also affect the survival of *L. casei* in intestinal juices (Table 5). The better tolerance of *Bifidobacteria* in the simulated intestinal environment compared to *Lactobacilli* could be due to a better capacity of *Bifidobacteria* to produce fibrils of exopolysaccharides, which have a protective effect, as well as a role in the adhesion to epithelial cells [42].

Despite the better survival of probiotic bacteria, especially *Bifidobacteria* and *Lactobacilli*, has been previously recorded as a result of the addition of Goji berry [13,43,44,45], the exact mechanism of stimulation or prophylactic action in gastric or intestinal juices is not thoroughly understood, and the distinct physicochemical properties of the foods/substrates in which Goji berry was previously added as a prebiotic, such as yogurt [43], Chinese pickles [44], and Goji berry juice [45] do not allow a clear answer to this question, as many factors such as protein and lipid content, microbial antagonism, etc. may also play a role in the growth and survival of probiotics. In our study, it appears that the concentration of Goji berry polysaccharides plays a significant role and is positively related to the stimulatory of prophylactic effect under stress conditions. More specifically, sample 3, which has the lowest amount of maltodextrin and highest amount of Goji berry extract and polysaccharide content, had the best overall prebiotic activity in both growth media and in gastric and intestinal juices, followed by sample 1, which had the second highest polysaccharide content. The beneficial effect of polysaccharides of *Lycium barbarum* and *Astragalus membranaceus* herb extracts in rats with acetic acid (AA)-induced ulcerative colitis have been previously documented by Zhao et al. [46], who linked the prophylactic effects of the plant extracts with a higher proliferation of epithelial cells and the promotion of intestinal barrier recovery after damage in vitro, as well as reduced mucosal damage, weight loss, and diarrhea in vivo. This therapeutic action may also involve improved survival and colonization of the gut by *Bifidobacteria*, and to a lesser extent *Lactobacilli*, in the presence of Goji berry polysaccharides. Furthermore, it is known that different monosaccharides from Goji berry polysaccharides can be used by lactic acid bacteria (LAB) [14] and thus enhance the production of lactic acid and other organic acids, resulting in the reduction of pH in the intestinal tissue. This also inhibits the growth of pathogenic microorganisms in the gut, causing significant structural cell membrane damage, protein denaturation, and the loss of enzyme functionality [47]. Previous studies have investigated the effect of various oligosaccharides, polysaccharides (including maltodextrin), and dietary fibers on the development of probiotic microorganisms, as well as on symbiotic formulations [48]. Kimoto-Nira et al. [48] suggest that the presence of certain sugars in the growth medium of probiotic bacteria positively affect their development, and thus probiotic action. According to the results presented in our study, the addition of the encapsulated sample 3 in the cultivation of *L. acidophilus* after 24 h caused a reduction of pH by ~0.5 compared with the control. However, pH reduction was not followed by a significant increase in the bacterial population. The possible explanation of this observation is that even if the bacterial population remains fairly stable, lactate production may be induced by the addition of encapsulated Goji berry extracts.

Vernazza et al. [49] showed that maltodextrin has limited effect on acid production and growth rate (h^−1^) of *B. Longum* Bb-46, whereas galactooligosaccharides (GOS), fructooligosaccharides (FOS), xylooligosaccharide (XOS), and glucose can increase the growth rate by 0.066, 0.027, 0.1040, and 0.0995 h^−1^, respectively. Also, a probiotic *L. plantarum* species was shown to produce glucomannan exopolysaccharides, utilizing different sugars, such as lactose, glucose, galactose, and sucrose [50]. Notably, a large concentration of galactose, as well as fructose and xylose oligosaccharides, are present in Goji berry polysaccharides [51,52], which may stimulate exopolysaccharide synthesis by probiotic bacteria and explain the stimulatory effect of Goji berry polysaccharides observed in our study. More specifically, the survival and prevalence of probiotic bacteria in the gastrointestinal tract is partly dependent on the existence of extracellular polysaccharides produced by certain LAB as well as by *Bifidobacteria* [41,51]. These polysaccharide capsules act as a “shield”, reducing the contact of the cells with the outer environment and facilitating survival under environmental stress conditions [42,51]. Interestingly, many of the building blocks of Goji berry polysaccharides, such as galactose, glucose, and mannose among others, are also components of the protective extracellular capsule that probiotic bacteria can synthesize [53]. Among 21 probiotic strains of *Bifidobacteria* (belonging to three different species) and Lactobacilli (belonging to four different species) isolated from human intestinal microbiota, it was found that the produced exopolysaccharides contained galactose and glucose in all biopolymers, and rhamnose in half of them, while mannose, fucose, and *N*-acetyl-glucosamine were also minor components in four *Bifidobacterium* and one *Lactobacillus* strains [52]. Therefore, it can be deduced that the utilization of Goji berry sugar moieties, and especially galactose, which is in high concentration both in Goji berry and probiotic bacteria polysaccharides, for building a protective exopolysaccharide layer in probiotic bacteria, may also be an alternative mechanism of prebiotic activity of Goji berry polysaccharides.

Also, the results of Giavasis et al. [54] showed that plant polyphenols (such as olive polyphenols) can have significant stimulatory effects upon the growth and lactate production of certain LAB, whether the polyphenols are encapsulated in maltodextrin or not. The results of the present study confirm these findings and the fact that maltodextrin is not an active prebiotic substance, since sample No 3 with only 2% maltodextrin was more efficient as prebiotic, compared to sample 2 (with 14% maltodextrin) and No 1 (with 7% maltodextrin). Also, the final encapsulated powder of sample 3 had the highest content of total polyphenols, as well total polysaccharides, (followed by sample 2, which had the second-best prebiotic effect), to which the comparatively better prebiotic activity could be attributed to. In fact, other studies have also shown that plant polyphenols can act (at low concentrations) as stimulants of LAB or probiotic bacteria in food (e.g., yogurt) [55,56] or in vivo [57]. Kafantaris et al. [57] reported that, when piglets were treated with grape pomace polyphenols, they had significantly lower levels of Enterobacteriaceae in their feces, and significantly increased levels of *Bifidobacterium* and *Lactobacillus* spp. In others studies conducted on rats [58] and humans [59], proanthocyanidin-rich extracts from grape seeds showed prophylactic effects against chemotherapy-induced intestinal injury (mucositis) [58] and significantly increased the number of *Bifidobacteria* (by 0.5 log cfu/g of feces on average), while inhibiting other groups such as Enterobacteriacae (0.8 log cfu/g of feces on average) [59].

Therefore, taking into account all the above results and previous studies, the prebiotic activity described for Goji berry extracts in this study may be a reflection of a combined dose-dependent effect of Goji berry polyphenols, which may have a stimulatory effect at certain (low) concentrations, and polysaccharides, which may either act directly as prophylactic agents, or be degraded into mono- and oligo-saccharides, which can be used as energy sources and especially building blocks for the biosynthesis of exopolysaccharides that have a protective role against the adverse gastrointestinal conditions.

## 4. Conclusions

In conclusion, the findings of this study demonstrate a species-specific in vitro stimulation and potential prebiotic activity of encapsulated aqueous extracts of Greek Goji berry fruits isolated by ultrasonic assisted extraction. These encapsulated extracts showed a clear stimulation of *B. lactis* Bb-12, *B. longum* Bb-46, *L. acidophilus*, and *L. casei* in terms of both increased growth and organic acid production in synthetic nutritive medium, while *L. rhamnosus* had enhanced lactate production after the addition of Goji berry extract, but demonstrated significantly improved growth in synthetic medium after the addition of Goji berry extract. With respect to cell viability under simulated gastric conditions, *B. lactis* had the highest viability, and all tested strains benefited from the addition of Goji berry extract, especially *B. longum,* which was sensitive to low pH but showed a strong prophylactic effect after the addition of sample 3, followed by *B. lactis* and *L. casei*, which also maintained a higher population when Goji berry extract (especially sample 3) was present. Under simulated intestinal conditions *B. longum* had the best survival rate among the three tested probiotic strains, and exhibited the highest prophylactic effect after addition of Goji berry sample 3, followed by *B. lactis*, while the simulated intestinal conditions were very lethal to the tested *L. casei* strain, which appeared to survive better for up to one hour in the presence of Goji berry extract, however its population diminished after three hours in intestinal juice.

Overall, the most effective prebiotic substance was sample No3 which had the least maltodextrin and the highest content of Goji berry polysaccharides and polyphenols, which may both have a role in the stimulatory and/or prophylactic effects towards the probiotic strains that were used in this study. Based on these results, encapsulated Goji berry aqueous extracts can be a candidate for development of novel prebiotic and symbiotic substances and could be utilized in food production to boost the proliferation and survival of probiotics in food and in the gastrointestinal tract. 

## Figures and Tables

**Table 1:** Concentration of Goji berry polyphenols and carbohydrates in liquid and dried-encapulated Goji berry extract (LGBE and DGBE, respectively), as well as maltodextrin or SiO2 concentration added in liquid Goji berry extracts (LGBE) before encapsulation of the three samples of dried-encapsulated Goji berry powder used in this study.

DGBE Sample	Goji Berry Carbohydrates (g/L) in LGBE	Goji Berry Polyphenols (mg/L) in LGBE	Maltodextrin or SiO2 Added in LGBE (g/L)	Goji Berry Carbohydrates % (*w/w*) in DGBE	Goji Berry Polyphenols % (*w/w*) in DGBE
1	26.9 ± 0.52	792 ± 2.2	70 (maltodextrin)	34.08 ± 0.33	0.74 ± 0.12
2	25.74 ± 1.07	756 ± 5.6	140 (maltodextrin)	19.69 ± 0.49	0.65 ± 0.11
3	28.24 ± 0.81	970 ± 6.4	20 (maltodextrin) + 10 (SiO2)	55.84 ± 0.52	0.94 ± 0.1

LGBE: Liquid Goji berry extract. DGBE: Dried (encapsulated) Goji berry extract.

**Table 2:** The pH evolution of synthetic growth medium inoculated with probiotic bacteria, without (control) or with the addition of encapsulated Goji berry extracts (samples 1–3).

		pH during Incubation in Growth Medium					
	0 h	8 h	24 h	34 h	Mean Delta pH at 8 h	Mean Delta pH at 24 h	Mean Delta pH at 34 h
*L. acidophilus control*	6.5 ± 0.02	5.5 ± 0.04 ^a^	4.92 ± 0.02 ^a^	3.83 ± 0.02 ^a^	0	0	0
*L. acidophilus* + sample 1	6.5 ± 0.01	5.43 ± 0.03 ^a^	4.74 ± 0.02 ^b^	3.65 ± 0.01 ^b^	0.07	0.18 *	0.18 *
L. *acidophilus* + sample 2	6.5 ± 0.02	5.49 ± 0.03 ^a^	4.83 ± 0.04 ^ab^	3.75 ± 0.03 ^ab^	0.01	0.09	0.08
*L. acidophilus*+ sample 3	6.5 ± 0.03	5.4 ± 0.02 ^a^	4.4 ± 0.02 ^c^	3.63 ± 0.01 ^b^	0.1	0.52 *	0.20 *
*L. casei control*	6.5 ± 0.02	5.19 ± 0.02 ^a^	4.11 ± 0.03 ^a^	3.96 ± 0.01 ^a^	0	0	0
*L. casei* + sample 1	6.5 ± 0.03	5.04 ± 0.03 ^b^	3.98 ± 0.02 ^a^	3.85 ± 0.02 ^a^	0.15 *	0.13	0.09
*L. casei* + sample 2	6.5 ± 0.01	5.09 ± 0.04 ^a^	4.01 ± 0.01 ^a^	3.85 ± 0.01 ^a^	0.10	0.10	0.09
*L. casei* + sample 3	6.5 ± 0.03	5.04 ± 0.02 ^b^	3.96 ± 0.02 ^b^	3.81 ± 0.02 ^b^	0.15 *	0.15 *	0.15 *
L. *rhamnosus control*	6.5 ± 0.02	5.2 ± 0.02 ^a^	3.95 ± 0.03 ^a^	3.93 ± 0.02 ^a^	0	0	0
*L. rhamnosus* + sample 1	6.5 ± 0.03	5.11 ± 0.04 ^a^	3.77 ± 0.03 ^bc^	3.75 ± 0.01 ^c^	0.09	0.18 *	0.18 *
*L. rhamnosus* + sample 2	6.5 ± 0.01	5.07 ± 0.06 ^a^	3.79 ± 0.02 ^b^	3.78 ± 0.01 ^b^	0.13	0.16 *	0.15 *
*L. rhamnosus* + sample 3	6.5 ± 0.01	5.19 ± 0.05 ^a^	3.76 ± 0.02 ^c^	3.75 ± 0.01 ^c^	0.01	0.19 *	0.18 *
*B. lactis* (Bb-12) *control*	6.5 ± 0.01	5.8 ± 0.02 ^a^	4.27 ± 0.04 ^a^	4.18 ± 0.03 ^a^	0	0	0
*B. lactis* (Bb-12) + sample 1	6.5 ± 0.02	5.66 ± 0.03 ^ab^	4.27 ± 0.05 ^a^	4.17 ± 0.04 ^a^	0.14	0	0.01
*B. lactis* (Bb-12) + sample 2	6.5 ± 0.02	5.67 ± 0.04 ^ab^	4.29 ± 0.04 ^a^	4.04 ± 0.01 ^ab^	0.13	−0.02	0.14 *
*B. lactis* (Bb-12) + sample 3	6.5 ± 0.03	5.65 ± 0.03 ^b^	4.23 ± 0.03 ^a^	4.02 ± 0.01 ^b^	0.15 *	0.04	0.16 *
*B. longum*(Bb-46) *control*	6.5 ± 0.02	5.86 ± 0.02 ^a^	4.62 ± 0.02 ^a^	4.45 ± 0.04 ^a^	0	0	0
*B. longum*(Bb-46) + sample 1	6.5 ± 0.03	5.73 ± 0.03 ^ab^	4.49 ± 0.01 ^ab^	4.23 ± 0.03 ^b^	0.13	0.13	0.22 *
*B. longum*(Bb-46) + sample 2	6.5 ± 0.01	5.64 ± 0.02 ^b^	4.41 ± 0.02 ^b^	4.25 ± 0.02 ^b^	0.22 *	0.21 *	0.2 *
*B. longum*(Bb-46) + sample 3	6.5 ± 0.01	5.78 ± 0.02 ^a^	4.29 ± 0.01 ^c^	4.11 ± 0.04 ^c^	0.08	0.33 *	0.34 *

Measurements were taken at 0 h (right after inoculation), 8, 25 and 34 h of incubation. Mean delta values represent differences of pH values compared to the control. Different letters in superscript (a–c) indicate differences in the mean values of the different samples within the same hour of incubation. Measurements that have a statistically significant difference to the control are highlighted by asterisk (*). The level of significance was set at *p* ≤ 0.05.

**Table 3:** Population (log cfu/mL) of probiotic bacteria in synthetic growth medium without (control) or with the addition of encapsulated Goji berry extracts (samples 1–3).

	Population (log cfu/mL) during Incubation in Growth Medium						
	0 h	8 h	24 h	34 h	Mean Delta log cfu/mL at 8 h	Mean Delta log cfu/mL at 24 h	Mean Delta log cfu/mL at 34 h
*L. acidophilus* control	4.93 ± 0.2	7.56 ± 0.3 ^a^	8.77 ± 0.4 ^a^	8.11 ± 0.2 ^b^	0	0	0
*L. acidophilus* + sample 1	4.93 ± 0.3	7.78 ± 0.2 ^a^	8.73 ± 0.6 ^a^	8.75 ± 0.1 ^a^	0.22	–0.04	0.64 *
*L. acidophilus* + sample 2	4.93 ± 0.1	7.56 ± 0.2 ^a^	8.71 ± 0.4 ^a^	8.61 ± 0.2 ^a^	0	–0.06	0.51 *
*L. acidophilus* + sample 3	4.93 ± 0.2	7.68 ± 0.1 ^a^	8.74 ± 0.4 ^a^	8.61 ± 0.2 ^a^	0.12	–0.03	0.5 *
*L. casei* control	6.85 ± 0.1	8.34 ± 0.2 ^a^	8.88 ± 0.2 ^a^	7.89 ± 0.2 ^b^	0	0	0
*L. casei* + sample 1	6.85 ± 0.2	8.49 ± 0.3 ^a^	9.04 ± 0.3 ^ab^	9.04 ± 0.1 ^a^	0.15	0.16	1.15 *
*L. casei* + sample 2	6.85 ± 0.3	8.39 ± 0.4 ^a^	9.04 ± 0.2 ^ab^	9.17 ± 0.1 ^a^	–0.1	0.16	1.13 *
*L. casei* + sample 3	6.85 ± 0.2	8.43 ± 0.5 ^a^	9.17 ± 0.1 ^ab^	9.23 ± 0.2 ^a^	0.09	0.29	1.34 *
*L. rhamnosus* control	5.86 ± 0.2	8.38 ± 0.2 ^a^	8.77 ± 0.2 ^a^	8.9 ± 0.1 ^a^	0	0	0
*L. rhamnosus* + sample 1	5.86 ± 0.2	8.43 ± 0.3 ^a^	8.88 ± 0.4 ^a^	8.87 ± 0.5 ^a^	0.05	0.11	–0.03
L. rhamnosus + sample 2	5.86 ± 0.1	8.41 ± 0.4 ^a^	8.84 ± 0.3 ^a^	8.86 ± 0.4 ^a^	0.03	0.07	–0.04
*L. rhamnosus* + sample 3	5.86 ± 0.3	8.38 ± 0.3 ^a^	8.89 ± 0.2 ^a^	8.9 ± 0.3 ^a^	0	0.12	0
*B. lactis* (Bb-12) control	3.99 ± 0.3	5.85 ± 0.2 ^a^	6.74 ± 0.2 ^a^	5.08 ± 0.2 ^c^	0	0	0
*B. lactis*(Bb-12) + sample 1	3.99 ± 0.2	5.88 ± 0.5 ^a^	6.82 ± 0.1 ^a^	6.83 ± 0.1 ^b^	0.03	0.08	1.75 *
*B. lactis*(Bb-12) + sample 2	3.99 ± 0.3	5.98 ± 0.4 ^a^	6.78 ± 0.2 ^a^	6.88 ± 0.2 ^b^	0.13	0.04	1.8 *
*B. lactis*(Bb-12) + sample 3	3.99 ± 0.1	6.08 ± 0.5 ^a^	6.84 ± 0.2 ^a^	7.08 ± 0.1 ^a^	0.23	0.1	2 *
*B. longum*(Bb-46) control	5.54 ± 0.3	6.46 ± 0.3 ^a^	7.87 ± 0.1 ^a^	7.85 ± 0.2 ^a^	0	0	0
*B. longum*(Bb-46) + sample 1	5.54 ± 0.2	6.49 ± 0.5 ^a^	7.98 ± 0.2 ^a^	8.08 ± 0.2 ^a^	0.03	0.11	0.23
*B. longum*(Bb-46) + sample 2	5.54 ± 0.2	6.47 ± 0.4 ^a^	7.89 ± 0.4 ^a^	7.91 ± 0.1 ^a^	0.01	0.02	0.06
*B.longum*(Bb-46) + sample 3	5.54 ± 0.1	6.38 ± 0.3 ^b^	7.99 ± 0.3 ^a^	8.11 ± 0.2 ^a^	–0.08	0.12	0.26

Measurements were taken at 0 h (right after inoculation), 8 h, 25 h, and 34 h of incubation. Mean delta values represent differences of population compared to the control. Different letters in superscript (a–c) indicate differences in the mean values of the different samples within the same hour of incubation. Measurements that have a statistically significant difference to the control are highlighted by asterisk (*). The level of significance was set at *p* ≤ 0.05.

**Table 4:** Population (log cfu/mL) of probiotic bacteria in simulated gastric juice medium without (control) or with the addition of encapsulated Goji berry extracts (samples 1–3).

	Population (log cfu/mL) during Incubation in Simulated Gastric Juice				
	0 h	1 h	3 h	Mean Delta log cfu/mL at 1 h	Mean Delta log cfu/mL at 3 h
*L, casei*	9.04 ± 0.3	8.14 ± 0.2 ^a^	7.57 ± 0.2 ^a^	0	0
*L, casei +* sample 1	9.04 ± 0.4	8.23 ± 0.3 ^a^	7.75 ± 0.3 ^a^	0.09	0.18
*L, casei +* sample 2	9.04 ± 0.2	8.16 ± 0.4 ^a^	7.65 ± 0.2 ^a^	0.02	0.08
*L, casei +* sample 3	9.04 ± 0.3	8.21 ± 0.3 ^a^	7.73 ± 0.4 ^a^	0.07	0.16
*B. lactis* (Bb-12®)	8.91 ± 0.2	8.32 ± 0.2 ^b^	7.17 ± 0.2 ^c^	0	0
*B. lactis*(Bb-12) + sample 1	8.91 ± 0.1	8.41 ± 0.4 ^b^	7.84 ± 0.2 ^b^	0.09	0.67 *
*B. lactis*(Bb-12) + sample 2	8.91 ± 0.2	8.39 ± 0.3 ^b^	7.8 ± 0.1 ^b^	0.07	0.63 *
*B. lactis*(Bb-12) + sample 3	8.91 ± 0.3	8.62 ± 0.3 ^ab^	8.15 ± 0.1 ^a^	0.3	0.98 *
*B, longum*(Bb-46)	8.84 ± 0.3	7.35 ± 0.2 ^c^	4.7 ± 0.2 ^c^	0	0
*B, longum*(Bb-46) *+* sample 1	8.84 ± 0.2	7.68 ± 0.1 ^b^	5.3 ± 0.3 ^b^	0.33 *	0.6 *
*B, longum*(Bb-46) *+* sample 2	8.84 ± 0.1	7.66 ± 0.1 ^b^	5.29 ± 0.2 ^b^	0.31 *	0.59 *
*B, longum*(Bb-46) *+* sample 3	8.84 ± 0.3	8.61 ± 0.3 ^a^	7.02 ± 0.4 ^a^	1.26 *	2.32 *

Measurements were taken at 0 h (right after inoculation), 1 h, and 3 h of incubation. Mean delta values represent differences of population compared to the control. Different letters in superscript (a–c) indicate differences in the mean values of the different samples within the same hour of incubation. Measurements that have a statistically significant difference to the control are highlighted by asterisk (*). The level of significance was set at *p* ≤ 0.05.

**Table 5:** Population (log cfu/mL) of probiotic bacteria in simulated intestinal juice medium without (control) or with the addition of encapsulated Goji berry extracts (samples 1–3).

	Population (log cfu/mL) during Incubation in Simulated Intestinal Juice				
	0 h	1 h	3 h	Mean Delta log cfu/mL at 1 h	Mean Delta log cfu/mL at 3 h
*L. casei*	9.04 ± 0.3	2.0 ± 0.6 ^b^	<1	0	ND
*L. casei* + sample 1	9.04 ± 0.4	3.5 ± 0.1 ^a^	<1	1.5 *	ND
*L. casei* + sample 2	9.04 ± 0.2	2.9 ± 0.2 ^a^	<1	0.9 *	ND
*L. casei* + sample 3	9.04 ± 0.3	3.8 ± 0.3 ^a^	<1	1.8 *	ND
*B. lactis* (Bb-12^®^)	8.91 ± 0.2	5.7 ± 0.3 ^b^	3.5 ± 0.1 ^b^	0	0
*B. lactis*(Bb-12^®^) + sample 1	8.91 ± 0.1	5.9 ± 0.2 ^ab^	3.84 ± 0.2 ^a^	0.2	0.34 *
*B. lactis*(Bb-12^®^) + sample 2	8.91 ± 0.2	5.8 ± 0.3 ^ab^	3.82 ± 0.1 ^a^	0.1	0.32 *
*B. lactis*(Bb-12^®^) + sample 3	8.91 ± 0.3	6.5 ± 0.2 ^a^	4.32 ± 0.3 ^a^	0.8 *	0.82 *
*B. longum*(Bb-46)	8.84 ± 0.3	6.99 ± 0.3 ^b^	5.45 ± 0.2 ^b^	0	0
*B. longum*(Bb-46) + sample 1	8.84 ± 0.2	7.58 ± 0.2 ^a^	6.41 ± 0.2 ^a^	0.59 *	0.96 *
*B. longum*(Bb-46) + sample 2	8.84 ± 0.1	7.6 ± 0.1 ^a^	6.39 ± 0.2 ^a^	0.61 *	0.94 *
*B. longum*(Bb-46) + sample 3	8.84 ± 0.3	7.93 ± 0.3 ^a^	6.61 ± 0.1 ^a^	0.94 *	1.16 *

Measurements were taken at 0 h (right after inoculation), 1 and 3 h of incubation. Mean delta values represent differences of population compared to the control. Different letters indicated differences in the means within the same hour of incubation. Measurements that have a statistically significant difference to the control are highlighted by asterisk (*). The level of significance was set at. *p* ≤ 0.05, ND: Not determined.
